# Sleep disturbance-related depressive symptom and brain volume reduction in shift-working nurses

**DOI:** 10.1038/s41598-020-66066-x

**Published:** 2020-06-04

**Authors:** Chang-hyun Park, Mirim Bang, Kook Jin Ahn, Woo Jung Kim, Na-Young Shin

**Affiliations:** 1 Department of Radiology, Seoul St. Mary’s Hospital, Catholic University of Korea College of Medicine, Seoul, Korea; 2 Center for Neuroprosthetics and Brain Mind Institute, Swiss Federal Institute of Technology (EPFL), Geneva, Switzerland; 3 Department of Psychiatry, Yongin Severance Hospital, Yonsei University College of Medicine, Yongin, Gyeonggi Korea; 4 Institute of Behavioral Sciences in Medicine, Yonsei University College of Medicine, Seoul, Korea; 5 Department of Psychiatry, Myongji Hospital, Hanyang University College of Medicine, Goyang, Gyeonggi Korea

**Keywords:** Predictive markers, Risk factors

## Abstract

Disturbed sleep is the most common effect of shift work. A large corpus of research indicates an association between sleep disturbance and depressive symptom in shift workers. In this study, we proposed the mediating role of grey matter (GM) structure in the relationship between sleep disturbance and depressive symptom. We collected structural MRI (sMRI) data as well as assessing the level of sleep disturbance and depressive symptom with the Pittsburgh Sleep disturbance Index and Zung Self-Rating Depression Scale, respectively, in 20 shift-working nurses and 19 day-working nurses. The shift-working nurses reported greater severity of sleep disturbance and depressive symptom, and furthermore, they exhibited reduced GM volume in the left postcentral gyrus (PostCG), right PostCG, right paracentral lobule, and left superior temporal gyrus (STG), compared to the day-working nurses. For each of the four brain regions, we formulated a mediation hypothesis by developing a mediation model that represents a causal chain between GM volume, sleep disturbance, and depressive symptom. Tests of the hypothesis on the mediation of GM volume revealed that inter-individual variations in left PostCG volume and left STG volume accounted for the influence of sleep disturbance on depressive symptom. These results suggest that structural alterations in PostCG and STG play an intervening role in the development of depressive symptom following sleep disturbance. We propose the need of considering neuroanatomical abnormalities in explaining and understanding symptomatic changes induced by sleep disturbance.

## Introduction

Due to the nature of shift work that involves a work schedule outside the traditional daytime work hours, it usually results in slow adaptation of the internal circadian clock to transitions of work schedules, that is, circadian desynchrony^1^. Though not fully certain^2^, circadian desynchrony may at least partially affect various health problems, not least disturbed sleep, exemplified as sleep interference during sleep hours and sleepiness during work hours^3^.

The level of sleep disturbance caused by shift work is similar to that observed in clinical insomnia^4^. Sleep disturbance could be a contributing factor to psychiatric problems^5^, and indeed, shift work and insomnia share the occurrence of psychiatric problems such as depression. As is well known for insomnia (for reviews, see^6,7^), shift work elevates levels of risk for depression^8–10^.

While neurobiological bases of the relationship between disturbed sleep and depression have not been fully elucidated, accumulating neuroimaging studies are contributing to better understanding of them^11,12^. In this study, we specifically employed structural MRI (sMRI) to examine the possible role of grey matter (GM) structure in the relationship since alterations in GM volume have been observed for depression (for a review, see^13^) as well as for disturbed sleep relevant to insomnia^14,15^, sleep debt^16^, and shift work^17^.

To address the question regarding the role of GM structure in the relationship between disturbed sleep and depression, we employed a mediation model, in which GM structure served as a mediator. This kind of intervening roles of GM structure have been suggested for the associations between different variable pairs, for instance, age and cognition^18^, family socioeconomic status and personality traits^19^, and adverse life events and antisocial behavior^20^.

We firstly checked the association of GM volume with both sleep disturbance and depressive symptom, and then we developed a mediation model to infer the mediating role of GM volume. Thus, the aims of the present study were (i) to replicate previous findings of the relationship between sleep disturbance and depressive symptom and their association with GM volume, and (ii) to analyze possible relationships between the three variables by focusing on the role of GM volume as the mediator between sleep disturbance and depressive symptom.

## Methods

### Participants

Participants included 20 shift-working (28.6 ± 3.2 years) and 19 day-working female nurses (30.8 ± 4.0 years) who met the following criteria: (i) aged between 20 and 49 years; and (ii) right-handed. In addition, the shift-working nurses were included when they have been experiencing shift work for more than six months, and the day-working nurses were included when they have not experienced shift work at all or more than two years have passed since they experienced shift work. Specifically, of different forms of shift work, the shift-working nurses were rotating shift workers in that their work schedule alternated between day, evening, and night. Participants who had signs of endocrine or psychiatric disorders, significant obstetric histories, or any contraindications to MRI scanning were excluded. We obtained written informed consent from all participants in accordance with the Declaration of Helsinki and its later amendments. The study was approved by the Institutional Review Board at the Seoul St. Mary’s Hospital, Seoul, Korea.

### Assessment of sleep disturbance and depressive symptom

In the participants, the level of sleep disturbance was assessed with the Pittsburgh Sleep Quality Index (PSQI)^21^, which is a self-report measure for assessing different aspects of sleep over the past month. A total score on 19 items of the PSQI, ranging from 0 to 21, represents the level of sleep disturbance, with a score higher than five being suggestive of significant sleep disturbance. In addition, the Zung Self-Rating Depression Scale (ZSDS)^22^ was used to assess the level of depressive symptom in the participants. It is a 20-item self-report measure for assessing different depressive symptoms. A total score ranges from 20 to 80, with a score of 45 and above being beyond the normal range.

We compared the PSQI and ZSDS between the shift-working nurses and day-working nurses by using the general linear model that included the participants’ age as a confounding variable. The statistical significance of group differences in the PSQI and ZSDS was determined at a *p* value of 0.05.

### Acquisition and processing of structural MRI data

MRI scans were collected using an Ingenia 3 T MRI system (Philips Healthcare, Best, Netherlands). Specifically, sMRI data were acquired in sagittal planes with a 3D T1-weighted turbo field echo imaging sequence: number of slices = 176, slice thickness = 1.00 mm, matrix size = 256 × 256, and in-plane resolution = 1.00 mm × 1.00 mm.

We used tools in CAT12 (http://www.neuro.uni-jena.de/cat/) to process the sMRI data. A brain image was segmented into probability maps of different tissue, including GM, whiter matter (WM), and corticospinal fluid. Each voxel in the GM probability map represented a local concentration of GM. The GM probability map was spatially registered to a reference brain in the standard space to eliminate inter-individual anatomy variations, and during the spatial normalization step, volume variations introduced due to spatial normalization were corrected by rescaling GM probability values in each voxel by Jacobian determinants derived from deformation fields. The spatially normalized GM probability map was then smoothed with an isotropic Gaussian kernel with 8 mm full-width-at-half-maximum.

### Comparison and correlation of GM volume

Of the 20 shift-working nurses, one for whom focal brain lesions were found and the other one for whom some PSQI items were omitted have been excluded. In addition, of the 19 day-working nurses, three were excluded due to problems in the acquisition or processing of sMRI data. A final sample size of 34, including 18 shift-working nurses (28.9 ± 3.2 years) and 16 day-working nurses (30.9 ± 4.2 years), were resultingly used for further analyses.

Voxel-wise comparisons of the GM probability maps were performed to detect GM volume changes in the shift-working nurses compared to the day-working nurses. The comparisons were made by employing the general linear model, as implemented in SPM12 (https://www.fil.ion.ucl.ac.uk/spm/software/spm12/), by adding the participants’ total intracranial volume (TIV) and age as confounding variables. Statistically significant differences in GM volume between the two groups were determined at an extent threshold of a *p* value of 0.05 family-wise error rate corrected for multiple comparisons with a height threshold of a *p* value of 0.001. For each cluster that exhibited statistical significant differences in GM volume between the two groups, we extracted GM volume as an average across voxels within the cluster, or more specifically, as an average weighed by each voxel’s coefficient estimate reflecting a relative group difference, from the participants’ GM probability maps.

In addition, voxel-wise correlations of the GM probability maps with the PSQI and ZSDS were conducted to check whether GM volume changes could be related to the levels of sleep disturbance and depressive symptom. Similar to the comparisons of the GM probability maps, the participants’ total TIV and age were used as confounding variables in the general linear model. Statistically significant correlations of GM volume with the PSQI and ZSDS were set at an extent threshold of a *p* value of 0.05 family-wise error rate corrected for multiple comparisons with a height threshold of a *p* value of 0.001.

### Assessment of relationships between GM volume, sleep disturbance, and depressive symptom

To assess relationships between GM volume, sleep disturbance, and depressive symptom, we included all participants in the two groups, rather than including the shift-working nurses only. We wanted to increase the sample size by combining the participants of the two groups, and more importantly, we supposed that the variables were continuously distributed across the two groups.

The relationship between sleep disturbance and depressive symptom and their association with GM volume were elucidated by establishing a mediation model. A mediation model based on a three-variable system involves two causal paths: the direct path from an independent variable (IV) to a dependent variable (DV) and the indirect path from the IV via a mediating variable (MV) to the DV^23^. Along the second causal path, the MV plays a mediating role to the extent that it accounts for the relationship between the IV and DV, given the following three conditions for testing the linkage of the mediation model are met: (i) the effect of the IV on the DV when regressing the DV on the IV (the total effect of the IV on the DV along both direct and indirect paths), (ii) the effect of the IV on the MV when regressing the MV on the IV (the effect of the IV on the MV along the indirect path), and (iii) the effect of the MV on the DV when regressing the DV on both the IV and MV (the effect of the MV on the DV along the indirect path).

In our setting of the three variables, sleep disturbance and depressive symptom were regarded as an IV and a DV, respectively, and GM volume was regarded as an MV. For GM volume extracted from each cluster of statistically significant group differences, we tested the three conditions. If the three conditions all held, the mediating role of GM volume was supposed when the effect of the IV (sleep disturbance) on the DV (depressive symptom) is less in the regression of the DV (depressive symptom) on both the IV (sleep disturbance) and MV (GM volume) than in the regression of the DV (depressive symptom) on the IV (sleep disturbance). That is, with respect to the impact of the IV (sleep disturbance) on the DV (depressive symptom), when the direct effect decreased considerably from the total effect, we supposed that the MV (GM volume) functioned as a mediator for the indirect effect. In these analyses, the participants’ TIV and age were covaried.

In addition, we tested the statistical significance of the indirect effect firstly by applying the Sobel test^24^ and secondly by employing the bootstrap approach^25^. Whereas the Sobel test is based on the assumption that the indirect effect is normally distributed, the bootstrap approach is a nonparametric method that relies on random sampling with replacement. For the Sobel test, we used a function included in the bda R package (https://cran.r-project.org/web/packages/bda/), and for the bootstrap approach, we used a function included in the mediation R package (https://cran.r-project.org/web/packages/mediation/). Specifically, we conducted 10,000 times of random sampling with replacement for the bootstrap approach. The statistical significance of the indirect effect was determined at a *p* value of 0.05 false discovery rate corrected for multiple comparisons for both the Sobel test and bootstrap approach.

## Results

### Differences in sleep disturbance and depressive symptom

The level of sleep disturbance, as assessed with the PSQI, was 7.1 ± 1.8 in the shift-working nurses and 4.4 ± 1.8 in the day-working nurses. The proportion of those who reported significant sleep disturbance (PSQI > 5) was 72% in the shift-working nurses and 31% in the day-working nurses. The level of depressive symptom, as assessed with the ZSDS, was 43.1 ± 6.4 in the shift-working nurses and 35.3 ± 4.9 in the day-working nurses. Whereas all the day-working nurses reported depressive symptom within the normal range, 39% of the shift-working nurses reported mild depression beyond the normal range (ZSDS ≥ 45).

**Figure 1:**
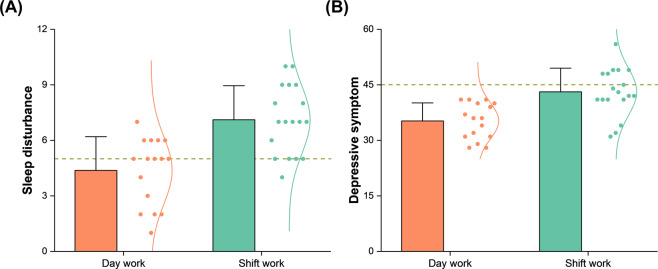
Statistically significant differences in (**A**) sleep disturbance and (**B**) depressive symptom between shift-working nurses and day-working nurses. A bar and an error bar, respectively, represent the mean and standard deviation of data points, which are represented as dots, along with a normal distribution curve fitted to them. A horizontal dotted line indicates a boundary above which significant sleep disturbance is supposed in (**A**) and depressive symptom beyond the normal range is supposed in (**B**).

When we compared sleep disturbance and depressive symptom between the shift-working nurses and day-working nurses, there were significant differences in both measures (Fig. 1). The shift-working nurses reported greater severity of sleep disturbance (*t*(31) = 4.1428, *p* = 0.0002) and depressive symptom (*t*(31) = 4.1132, *p* = 0.0003) than the day-working nurses did.

### Comparison and correlation of GM volume

**Figure 2:**
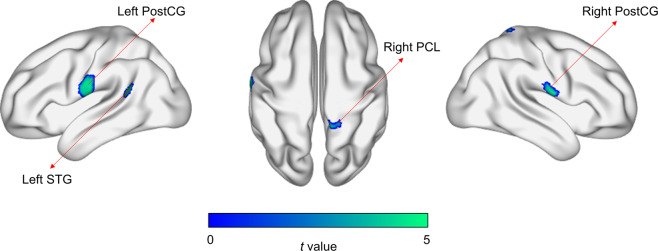
Statistically significant differences in grey matter volume between shift-working nurses and day-working nurses. Four clusters of statistically significant differences apply to the left postcentral gyrus (PostCG), right PostCG, right paracentral lobule (PCL), and left superior temporal gyrus (STG).

When we made voxel-wise comparisons of GM volume between the two groups, statistically significant reductions in GM volume were seen in four clusters, which corresponded to the left postcentral gyrus (PostCG), right PostCG, right paracentral lobule (PCL), and left superior temporal gyrus (STG) (Fig. 2 and Table 1). Besides, when we assessed voxel-wise correlations of GM volume with the PSQI and ZSDS, statistically significant negative correlations between GM volume in the left PostCG and the PSQI and between GM volume in the left STG and the ZSDS were seen when we considered the two groups together (Fig. S1 and Table S1).

**Table 1:** Clusters of statistically significant differences in grey matter volume between shift-working nurses and day-working nurses.

No	Extent (voxel count)	Brain region	Peak voxel		
			*t* value	*p* value	Coordinates (*x*, *y*, *z* in mm)
1	1248	Left PostCG	5.0605	<0.0001	−71, −2, 23
2	590	Right PostCG	5.3099	<0.0001	74, −5, 15
3	421	Right PCL	5.9702	<0.0001	12, −56, 83
4	285	Left STG	6.2892	<0.0001	−53, −47, 18

PostCG, postcentral gyrus; PCL, paracentral lobule; and STG, superior temporal gyrus.

### Relationships between GM volume, sleep disturbance, and depressive symptom

Regarding the first condition for establishing the mediation model, the association of sleep disturbance with depressive symptom (*t*(30) = 3.3740, *p* = 0.0021) was observed (Fig. S2), such that more disturbed sleep predicted more severe depressive symptom. For the second condition, more severe sleep disturbance was related to greater reductions in GM volume over the left PostCG (*t*(30) = −3.1399, *p* = 0.0038), right PostCG (*t*(30) = −3.6766, *p* = 0.0009), right PCL (*t*(30) = −2.5833, *p* = 0.0149), and left STG (*t*(30) = −3.1953, *p* = 0.0033) (Fig. S3). For the third condition, more severe depressive symptom was predicted, after adjusting for inter-individual differences in sleep disturbance, from greater GM volume reductions in the left PostCG (*t*(29) = −2.3998, *p* = 0.0230) and left STG (*t*(29) = −3.8116, *p* = 0.0007), but not in the right PostCG (*t*(29) = −0.5548, *p* = 0.5833) and right PCL (*t*(29) = −1.6160, *p* = 0.1169) (Fig. S4). Statistics for these three conditions are summarized in Table 2.

**Table 2:** Statistics of three conditions for establishing a mediation model that describes how the influence of an independent variable (IV, sleep disturbance) on a dependent variable (DV, depressive symptom) is intervened by a mediating variable (MV, grey matter volume).

Condition	Regression	Effect	Brain region	*t* value	*p* value
1	DV ~ IV	IV on DV		3.3740	0.0021*
2	MV ~ IV	IV on MV	Left PostCG	−3.1399	0.0038*
			Right PostCG	−3.6766	0.0009*
			Right PCL	−2.5833	0.0149*
			Left STG	−3.1953	0.0033*
3	DV ~ IV + MV	MV on DV	Left PostCG	−2.3998	0.0230*
			Right PostCG	−0.5548	0.5833
			Right PCL	−1.6160	0.1169
			Left STG	−3.8116	0.0007*

PostCG, postcentral gyrus; PCL, paracentral lobule; STG, superior temporal gyrus; and *, statistical significance.

**Figure 3:**
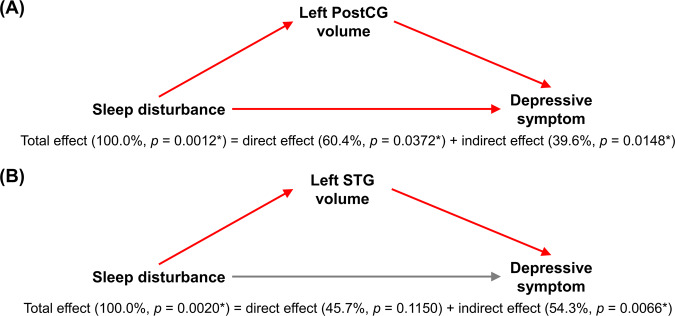
Mediation models that describe the mediation of (A) left postcentral gyrus (PostCG) volume and (B) left superior temporal gyrus (STG) volume in the relationship between sleep disturbance and depressive symptom. A link between a pair of variables is indicated in red when the respective linear relation is statistically significant. *, statistical significance.

While the total effect of sleep disturbance on depressive symptom was statistically significant, the direct effect between the two variables after adjusting for inter-individual differences in GM volume was not statistically significant for the left PostCG (*t*(29) = 1.9573, *p* = 0.0600) and left STG (*t*(29) = 1.5898, *p* = 0.1227) that satisfied all the three conditions above. That is, the intervening role of GM volume in the relationship between sleep disturbance and depressive symptom could be supposed for the two brain regions. In formal tests of the mediation hypothesis, the indirect effect was statistically significant for both the left PostCG (*z* = 2.0268, *p* = 0.0427) and left STG (*z* = 2.5191, *p* = 0.0118) according to the Sobel test. Besides, according to the bootstrap approach, the indirect effect was statistically significant for both the left PostCG (*p* = 0.0148) and left STG (*p* = 0.0066), but the direct effect was not statistically significant for the left STG only (*p* = 0.1150). Statistics for these tests of the mediation model are summarized in Fig. 3 and Table 3.

**Table 3:** Statistics of tests for the mediation of a mediating variable (grey matter volume) in the relationship between an independent variable (sleep disturbance) and a dependent variable (depressive symptom).

Brain region	Sobel test			Bootstrap approach					
	Indirect effect			Total effect		Direct effect		Indirect effect	
	Effect size	*z* value	*p* value	Effect size	*p* value	Effect size	*p* value	Effect size	*p* value
Left PostCG	0.6366	2.0268	0.0427*	1.6077	0.0012*	0.9711	0.0372*	0.6366	0.0148*
Left STG	0.8731	2.5191	0.0118*	1.6077	0.0020*	0.7347	0.1150	0.8731	0.0066*

PostCG, postcentral gyrus; STG, superior temporal gyrus; and *, statistical significance.

## Discussion

In this study, we replicated previous findings of reduced GM volume as well as greater severity of sleep disturbance and depressive symptom in shift workers compared to day workers. By developing a mediation model, furthermore, we revealed relationships between GM volume, sleep disturbance, and depressive symptom, such that GM volume in the left PostCG and left STG mediated the impact of sleep disturbance on depressive symptom.

Because of its leading to circadian rhythm disruption, shift work brings about disturbed sleep, which is the most common of many health-related effects of shift work. Although the shift-working nurses reported more severe sleep disturbance than the day-working nurses did, about one third of the day-working nurses reported significant sleep disturbance, indicating sleep problems even in healthy individuals^26,27^. Since the association between disturbed sleep and depression appears not to be limited to individuals with clinical sleep problems^28,29^, we supposed that the influence of disturbed sleep on depression could be examined for all participants in the two groups.

Voxel-wise comparisons showed that GM volume in the left PostCG, right PostCG, right PCL, and left STG was reduced in the shift-working nurses compared to the day-working nurses. The underlying mechanism remains unclear, but GM atrophy induced by shift work could be still described as neuronal loss and dysfunctional connections between neurons. As shown in sleep-restricted animals, disturbed sleep could have a negative impact on neuronal regeneration by acting as a stressor^30,31^. Furthermore, as exhibited with diffusion weighted MRI, WM alterations specifically related to demyelination, as indicated by increased radial diffusivity^32^, may have led to impaired neuronal connections.

Voxel-wise and cluster-wise correlations showed that GM volume specifically in the left PostCG and left STG was related to symptomatic changes. Structural abnormalities in the PostCG and STG could have been seen in both sleep disorder^15^ and major depressive disorder^33^, although causal links between brain structural alterations and symptomatic changes may not be inferred in those. As both the PostCG and STG are involved in the processing of external stimuli, abnormal processing in them and its representation as the form of GM atrophy may precede or follow from the development of sleep disturbance and depressive symptom. In particular, the involvement of the PostCG in the process of falling asleep^34^ and the role of the STG for emotional processing^35^ could imply the relevance of their morphologic changes to sleep disturbance and depressive symptom, respectively. In this study, it is unclear why only the left of the two PostCGs has been observed to be related to sleep disturbance, and we suppose a lack of statistical power for the right PostCG since its relationship with sleep disturbance has been described before^15^.

In the relationship between disturbed sleep and depression, we postulated a possible intervening role of GM structure, and we attempted to delineate relationships between GM volume, sleep disturbance, and depressive symptom by developing a mediation model that presumes direct and indirect paths between sleep disturbance and depressive symptom and the mediating role of GM volume along the indirect path. In the mediation model, the total effect of sleep disturbance on depressive symptom was assumed to be composed of the direct effect along the direct path and the indirect effect along the indirect path. While the total effect was statistically significant, the indirect effect was statistically significant for the mediators of left PostCG volume and left STG volume. Thus, we suppose that the mediation of GM volume in the left PostCG and left STG could account for considerably the influence of sleep disturbance on depressive symptom. That is, reduced GM volume in the PostCG and STG is regarded as serving as a contributory causal factor in the development of depressive symptom following sleep disturbance.

Given a developed mediation model, the degree of mediation may be interpreted as being complete or partial when the indirect effect is statistically significant, despite the suggestion on the needlessness of such a claim^36^. The conclusion of complete mediation may be reliant on whether the direct effect is not statistically significant, or more strongly, whether the direct effect is zero^23^. For the mediation of left PostCG volume, the direct effect was statistically significant according to the bootstrap approach, so that it is considered partial mediation. For the mediation of left STG volume, only 56% of the total effect was explained by the indirect effect despite the lack of statistical significance of the direct effect, so that it could be claimed as partial mediation as well.

In developing a mediation model, relationships between GM volume, sleep disturbance, and depressive symptom may be explained by different assumptions about a causal chain between the three variables. We suppose that the proposed causal chain is preferable when the following understandings are taken into account. Firstly, the causal influence of sleep disturbance on depressive symptom is suggested based on the understanding that disturbed sleep is the most prominent direct impact of shift work. Although it is obvious from the literature that there is a relationship between disturbed sleep and depression^7^, the direction of influences between them seems to be complicated in general circumstances; it has been suggested that the relationship could be bi-directional^7^ or they may be comorbid with each other^37^. In shift workers, nevertheless, the temporal precedence of disturbed sleep in the development of depression appears to be clear when we consider the mechanism of sleep disturbance in relation to circadian rhythms^4^.

Secondly, regarding the causal influence of GM volume on depressive symptom, we assumed the mediating role of GM structure in the occurrence of symptomatic changes, following mediational chains assumed in previous studies^38–41^. However, this directional influence is may not be assured since consistent changes in GM structure are also supposed to be caused by depression^13^. With respect to the occurrence of GM structural changes and depressive symptom following sleep disturbance, it is notable that both of them have been usually found at the chronic phase of sleep disturbance^6,15,16^. To capture the temporal order of the occurrence of GM structural changes and depressive symptom in chronic sleep disturbance, future investigations based on longitudinal assessments of them would be warranted.

Although the relationship between disturbed sleep and depression has been well recognized in many disorders, neurobiological underpinnings of the relationship are yet to be further investigated. We suppose shift workers could be appropriate subjects to study the relationship, since shift work-induced disruption to circadian rhythms is known as the underlying cause of the occurrence of subsequent symptomatic changes. Based on the clear causal influence of disturbed sleep on depression in shift workers, the mediating roles of GM function^12^ and herein GM structure in the relationship could be suggested. We propose an investigation into causal chains between brain function and structure, sleep disturbance, and depressive symptom in shift workers could help to better understand the relationship between disturbed sleep and depression and thus to establish therapeutic strategies towards symptomatic improvement, for instance, in a way that decides target sites for brain stimulation in remediating disturbed sleep-induced deficits^42^.

We should comment on some limitations of the current work. Most of all, since we included all participants to develop a mediation model, the intervening role of GM volume may not be intrinsically linked to shift work. Our findings are more suitably about relationships between GM volume, sleep disturbance, and depression not limited to shift workers. Secondly, in relation to the first limitation, this investigation is based on a relatively small sample size, so that a validation study with a larger sample size is required. Thirdly, the mediation model that we have developed might not conclusively imply the mediating role of GM volume because such a mediation model based on a three-variable system is a relatively simple model in which complex relationships between more diverse variables have not been fully considered. Indeed, sleep disturbance and depressive symptom could be associated with fatigue^43^, stress^44^, and anxiety^45^ as well, so that a larger causal model that includes such additional variables would provide a more complete view on the relationships.

In sum, we demonstrated the relationship between sleep disturbance and depressive symptom and their association with GM volume in shift workers and day workers, in accord with previous studies. Furthermore, we revealed relationships between GM volume, sleep disturbance, and depressive symptom by proposing the intervening role of GM volume in the impact of sleep disturbance on depressive symptom. These findings suggest the need of considering neuroanatomical abnormalities in explaining symptomatic changes caused by sleep disturbance such as in shift workers. We suppose this investigation would be a step forward in the better understanding of neuroanatomical and symptomatic changes related to shift work.

## Supplementary information

Supporting Information.
