# Supplementary material for: Sleep disturbance-related depressive symptom and brain volume reduction in shift-working nurses
Source: Sci Rep. 2020 Jun 4;10:9100. doi: 10.1038/s41598-020-66066-x (PMC7272417; doi:10.1038/s41598-020-66066-x)
Supplement: Supplementary file 1 — Supporting Information. [file 41598_2020_66066_MOESM1_ESM.pdf]

***Supporting Information*:**

Sleep disturbance-related depressive symptom and brain volume reduction in shift-working nurses

Chang-hyun Park[1,2], Mirim Bang[1], Kook Jin Ahn[1], Woo Jung Kim[3,4,5,*], Na-Young Shin[1,*]

[1] Department of Radiology, Seoul St. Mary's Hospital, Catholic University of Korea College of Medicine, Seoul, Korea
[2] Center for Neuroprosthetics and Brain Mind Institute, Swiss Federal Institute of Technology (EPFL), Geneva, Switzerland
[3] Department of Psychiatry, Yongin Severance Hospital, Yonsei University College of Medicine, Yongin, Gyeonggi, Korea
[4] Institute of Behavioral Sciences in Medicine, Yonsei University College of Medicine, Seoul, Korea
[5] Department of Psychiatry, Myongji Hospital, Hanyang University College of Medicine, Goyang, Gyeonggi, Korea

* Na-Young Shin. nyshin@catholic.ac.kr; Woo Jung Kim. woojay00@gmail.com

Supporting Figures and Tables

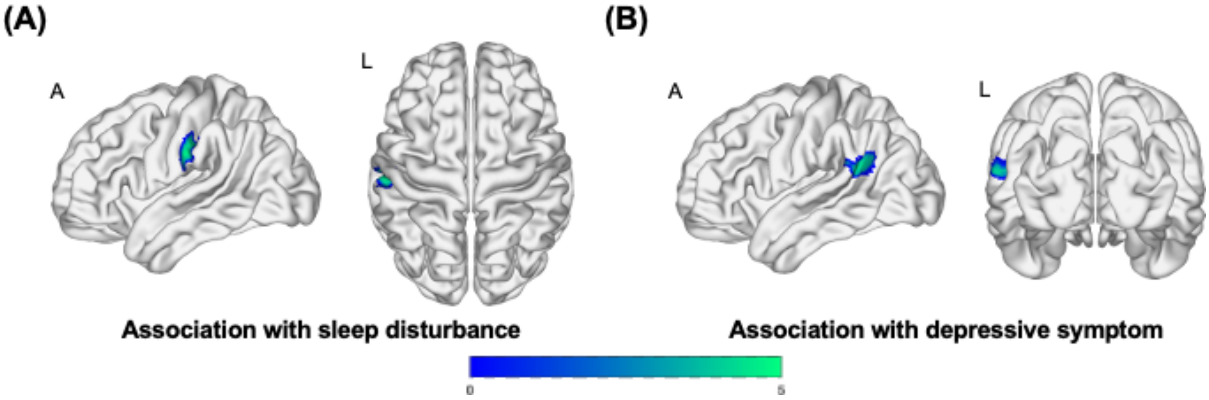

Figure S1. Statistically significant correlations of grey matter volume with (A) sleep disturbance and (B) depressive symptom. Clusters of statistically significant correlations apply to the left postcentral gyrus in (A) and the left superior temporal gyrus in (B). A, anterior; and L, left.

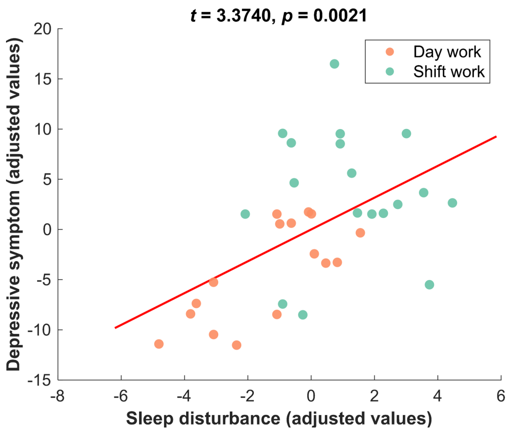

Figure S2. The association of sleep disturbance with depressive symptom. A line in red represents a statistically significant linear fit to data points.

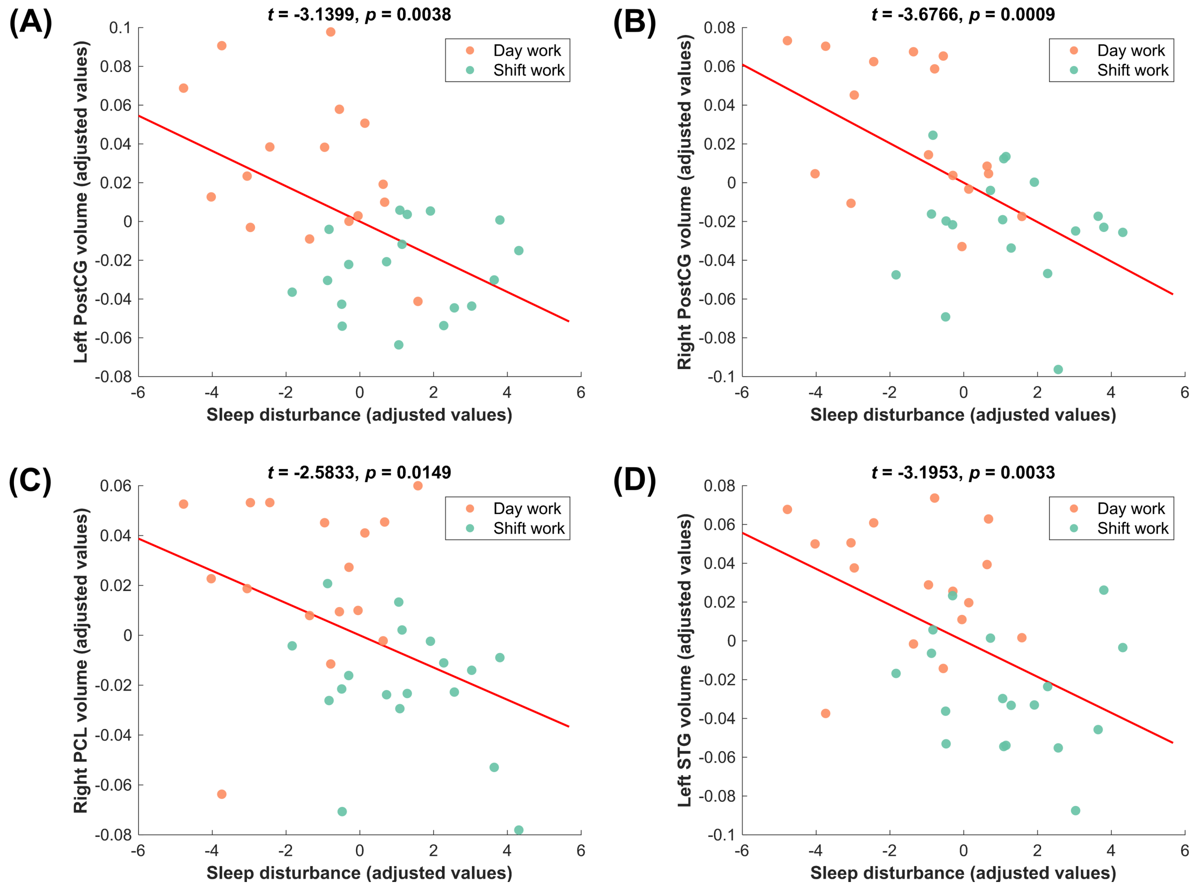

Figure S3. The association of sleep disturbance with (A) left postcentral gyrus (PostCG) volume, (B) right PostCG volume, (C) right paracentral lobule (PCL) volume, and (D) left superior temporal gyrus (STG) volume. A line in red represents a statistically significant linear fit to data points.

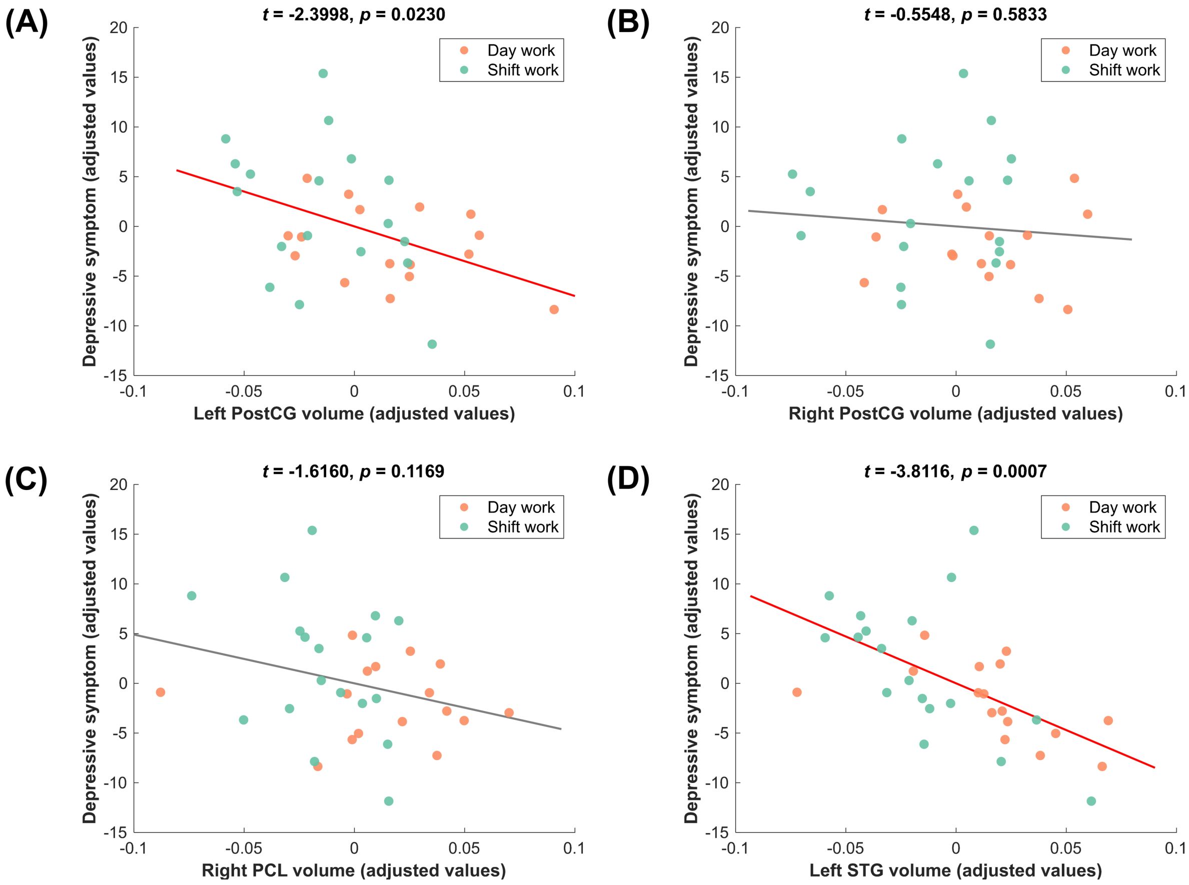

Figure S4. The association of (A) left postcentral gyrus (PostCG) volume, (B) right PostCG volume, (C) right paracentral lobule (PCL) volume, and (D) left superior temporal gyrus (STG) volume with depressive symptom, after adjusting for inter-individual differences in sleep disturbance. Lines in red and grey, respectively, represent statistically significant and non-significant linear fits to data points.

Table S1. Clusters of statistically significant correlations of grey matter volume with (A) sleep disturbance and (B) depressive symptom.

(A)

No	Extent (voxel count)	Brain region	Peak voxel		
			t value	*p* value	Coordinates (*x*, *y*, *z* in mm)
1	729	Left PostCG	5.7017	< 0.0001	-54, -15, 28

(B)

No	Extent (voxel count)	Brain region	Peak voxel		
			t value	*p* value	Coordinates (*x*, *y*, *z* in mm)
1	990	Left STG	5.8053	< 0.0001	-58, -51, 18

PostCG, postcentral gyrus; and STG, superior temporal gyrus.
